# Supplementary material for: Freshwater Mussels Show Elevated Viral Richness and Intensity during a Mortality Event
Source: Viruses. 2022 Nov 23;14(12):2603. doi: 10.3390/v14122603 (PMC9785814; doi:10.3390/v14122603)
Supplement: Supplementary file 1 [file viruses-14-02603-s001.zip › Supplemental Figure S2 Mucket Virus Phylogenies.pdf]

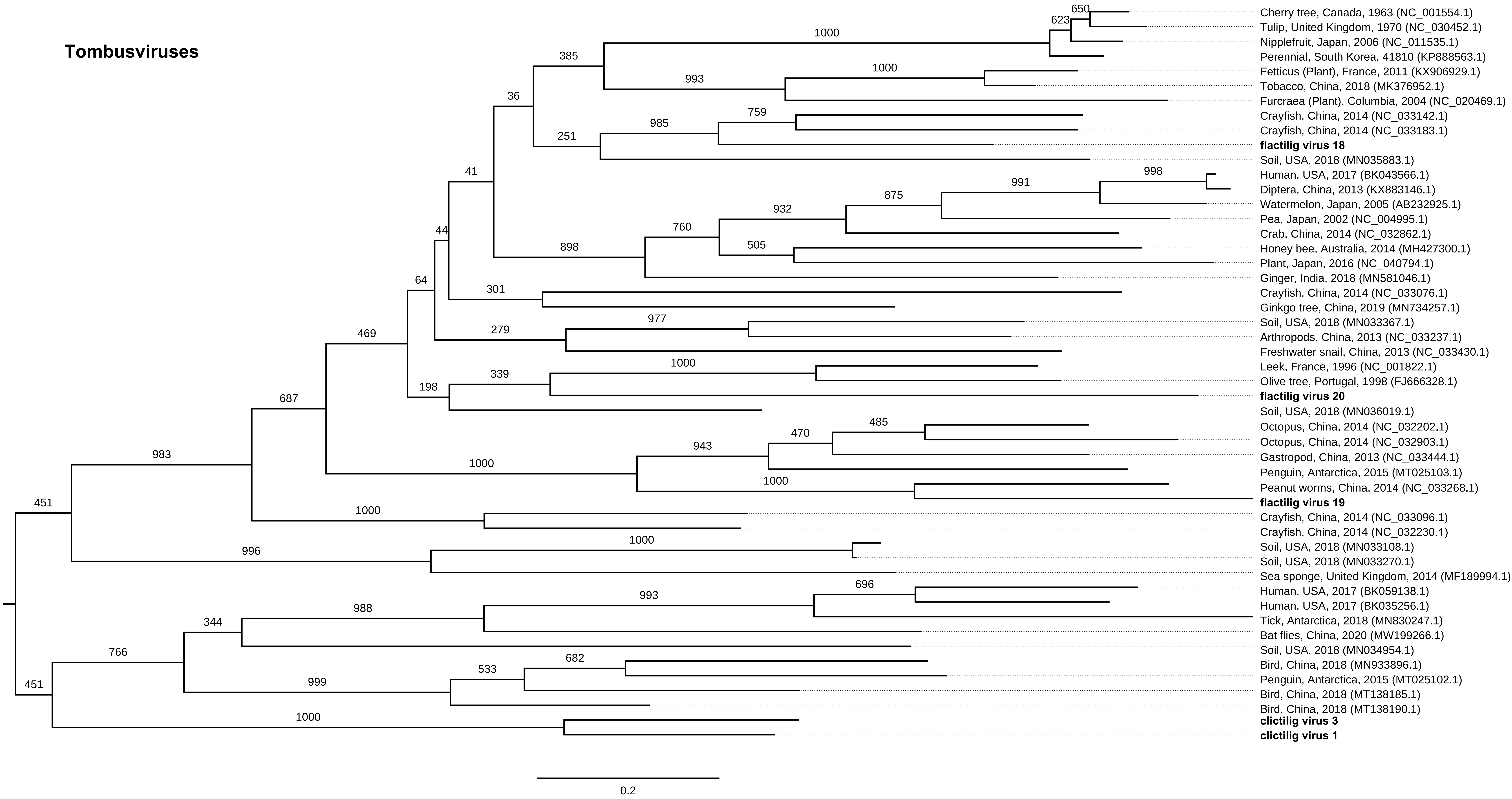
Tombusviruses
Cherry tree, Canada, 1963 (NC_001554.1)
Tulip, United Kingdom, 1970 (NC_030452.1)
Nipplefruit, Japan, 2006 (NC_011535.1)
Perennial, South Korea, 41810 (KP888563.1)
Fetticus (Plant), France, 2011 (KX906929.1)
Tobacco, China, 2018 (MK376952.1)
Furcraea (Plant), Columbia, 2004 (NC_020469.1)
Crayfish, China, 2014 (NC_033142.1)
Crayfish, China, 2014 (NC_033183.1)
flactilig virus 18
Soil, USA, 2018 (MN035883.1)
Human, USA, 2017 (BK043566.1)
Diptera, China, 2013 (KX883146.1)
Watermelon, Japan, 2005 (AB232925.1)
Pea, Japan, 2002 (NC_004995.1)
Crab, China, 2014 (NC_032862.1)
Honey bee, Australia, 2014 (MH427300.1)
Plant, Japan, 2016 (NC_040794.1)
Ginger, India, 2018 (MN581046.1)
Crayfish, China, 2014 (NC_033076.1)
Ginkgo tree, China, 2019 (MN734257.1)
Soil, USA, 2018 (MN033367.1)
Arthropods, China, 2013 (NC_033237.1)
Freshwater snail, China, 2013 (NC_033430.1)
Leek, France, 1996 (NC_001822.1)
Olive tree, Portugal, 1998 (FJ666328.1)
flactilig virus 20
Soil, USA, 2018 (MN036019.1)
Octopus, China, 2014 (NC_032202.1)
Octopus, China, 2014 (NC_032903.1)
Gastropod, China, 2013 (NC_033444.1)
Penguin, Antarctica, 2015 (MT025103.1)
Peanut worms, China, 2014 (NC_033268.1)
flactilig virus 19
Crayfish, China, 2014 (NC_033096.1)
Crayfish, China, 2014 (NC_032230.1)
Soil, USA, 2018 (MN033108.1)
Soil, USA, 2018 (MN033270.1)
Sea sponge, United Kingdom, 2014 (MF189994.1)
Human, USA, 2017 (BK059138.1)
Human, USA, 2017 (BK035256.1)
Tick, Antarctica, 2018 (MN830247.1)
Bat flies, China, 2020 (MW199266.1)
Soil, USA, 2018 (MN034954.1)
Bird, China, 2018 (MN933896.1)
Penguin, Antarctica, 2015 (MT025102.1)
Bird, China, 2018 (MT138185.1)
Bird, China, 2018 (MT138190.1)
clictilig virus 3
clictilig virus 1
0.2

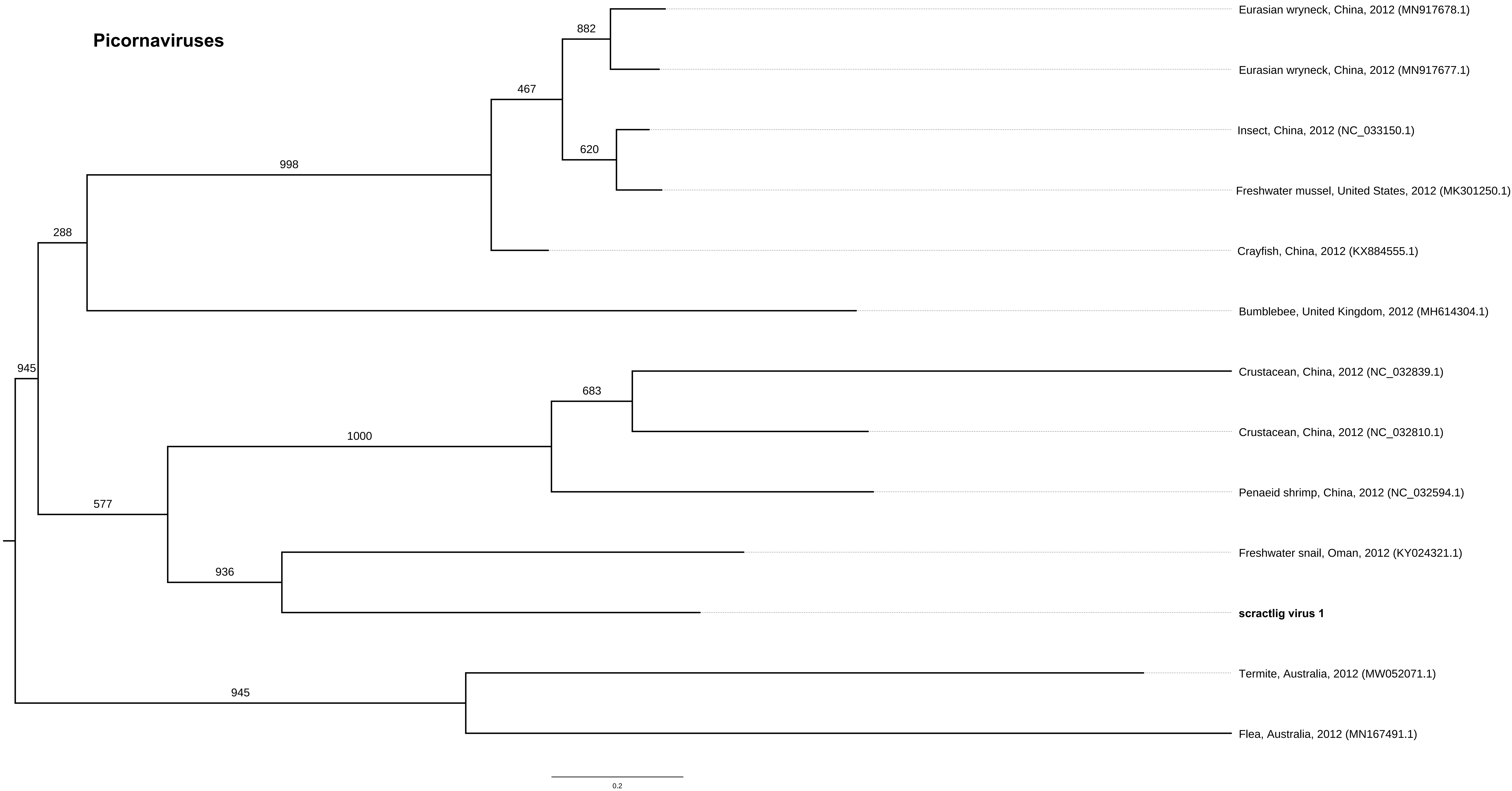

Picornaviruses
Eurasian wryneck, China, 2012 (MN917678.1)
Eurasian wryneck, China, 2012 (MN917677.1)
Insect, China, 2012 (NC_033150.1)
Freshwater mussel, United States, 2012 (MK301250.1)
Crayfish, China, 2012 (KX884555.1)
Bumblebee, United Kingdom, 2012 (MH614304.1)
Crustacean, China, 2012 (NC_032839.1)
Crustacean, China, 2012 (NC_032810.1)
Penaeid shrimp, China, 2012 (NC_032594.1)
Freshwater snail, Oman, 2012 (KY024321.1)
scractlig virus 1
Termite, Australia, 2012 (MW052071.1)
Flea, Australia, 2012 (MN167491.1)
882
467
620
998
288
945
683
1000
577
936
945
0.2

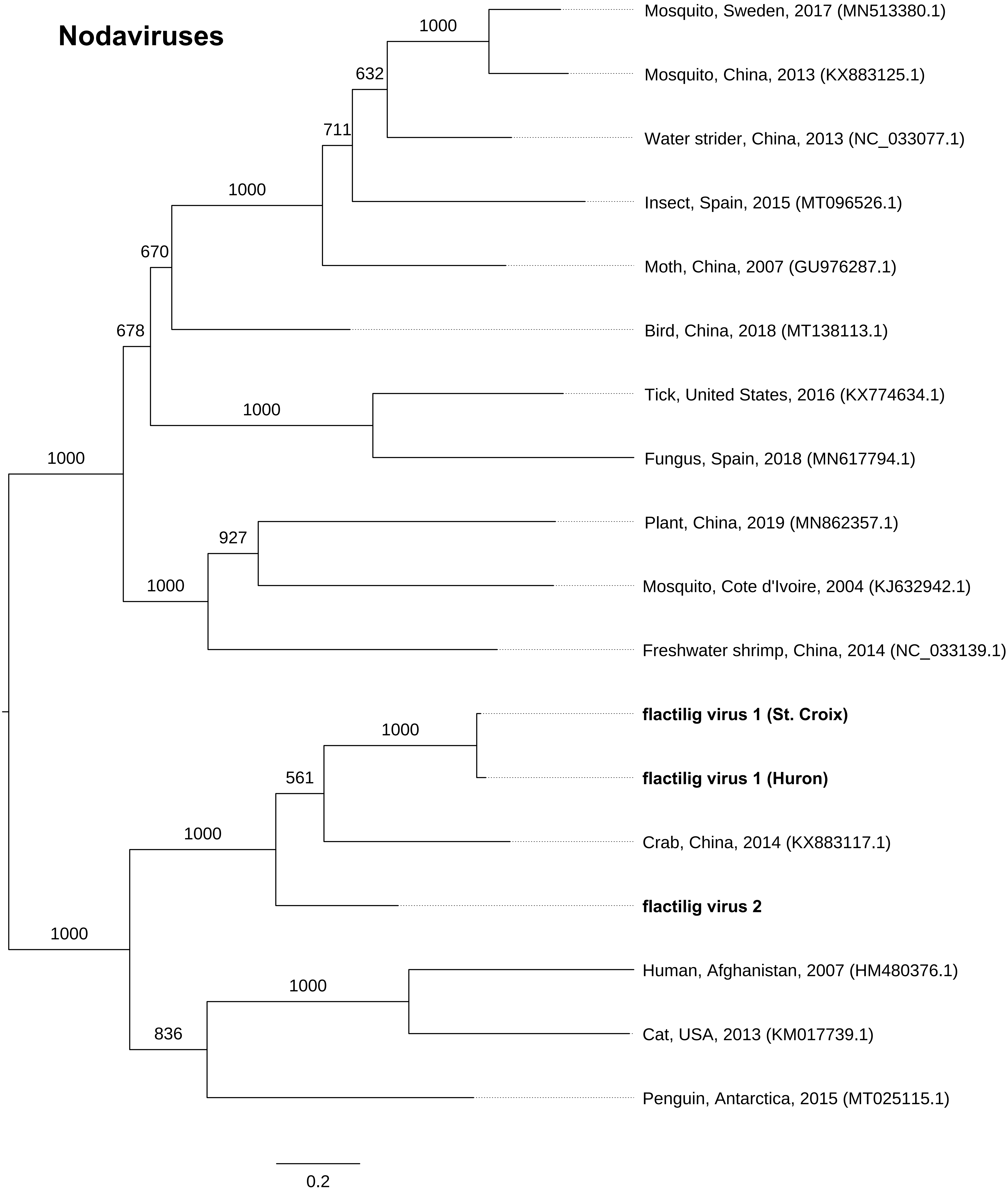
Nodaviruses
1000
Mosquito, Sweden, 2017 (MN513380.1)
632
Mosquito, China, 2013 (KX883125.1)
711
Water strider, China, 2013 (NC_033077.1)
1000
Insect, Spain, 2015 (MT096526.1)
670
Moth, China, 2007 (GU976287.1)
678
Bird, China, 2018 (MT138113.1)
1000
Tick, United States, 2016 (KX774634.1)
1000
Fungus, Spain, 2018 (MN617794.1)
927
Plant, China, 2019 (MN862357.1)
1000
Mosquito, Cote d'Ivoire, 2004 (KJ632942.1)
Freshwater shrimp, China, 2014 (NC_033139.1)
1000
flactilig virus 1 (St. Croix)
561
flactilig virus 1 (Huron)
1000
Crab, China, 2014 (KX883117.1)
flactilig virus 2
1000
1000
Human, Afghanistan, 2007 (HM480376.1)
836
Cat, USA, 2013 (KM017739.1)
Penguin, Antarctica, 2015 (MT025115.1)
0.2

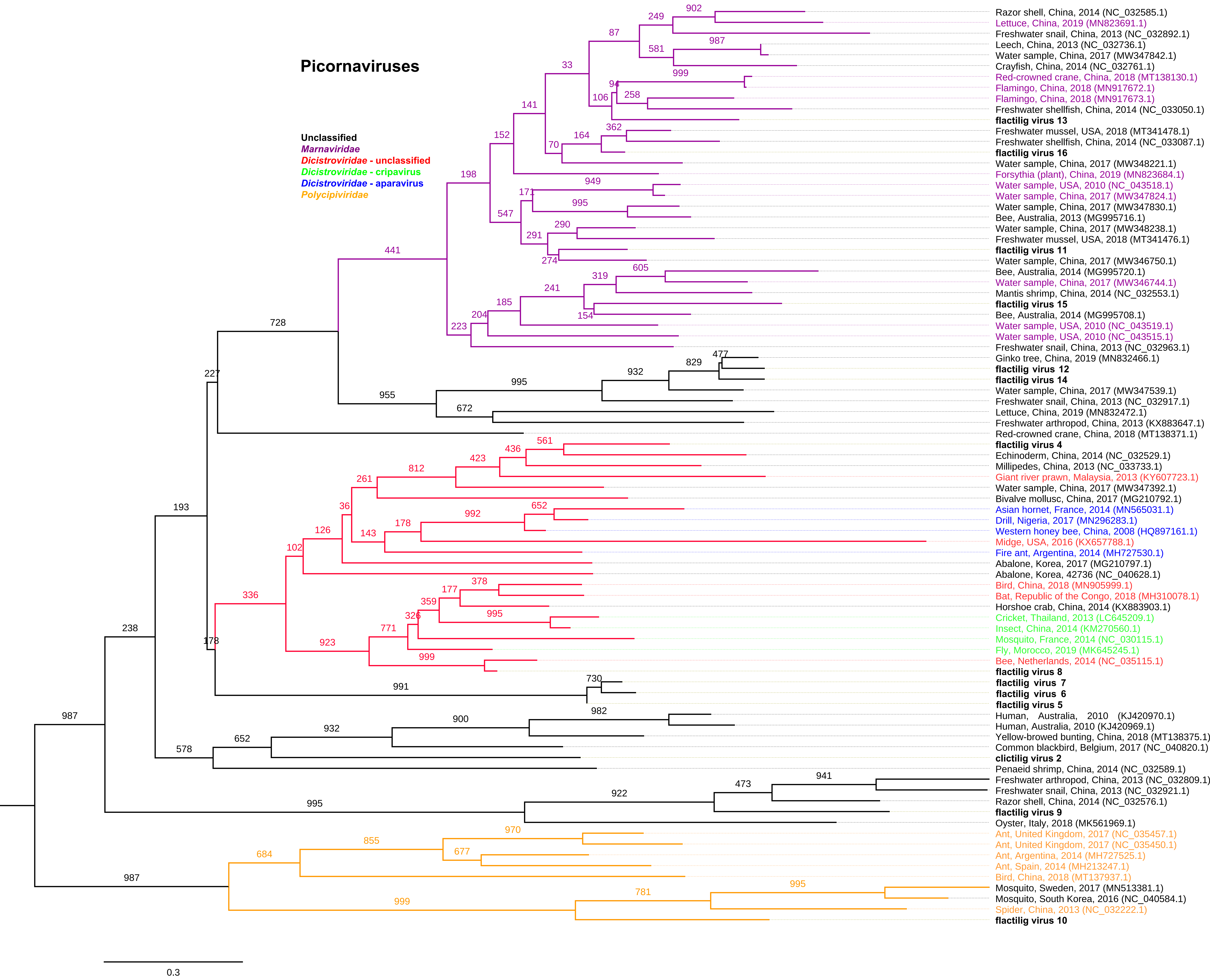

Picornaviruses
Unclassified
Marnaviridae
Dicistroviridae - unclassified
Dicistroviridae - cripavirus
Dicistroviridae - aparavirus
Polycipiviridae
Razor shell, China, 2014 (NC_032585.1)
Lettuce, China, 2019 (MN823691.1)
Freshwater snail, China, 2013 (NC_032892.1)
Leech, China, 2013 (NC_032736.1)
Water sample, China, 2017 (MW347842.1)
Crayfish, China, 2014 (NC_032761.1)
Red-crowned crane, China, 2018 (MT138130.1)
Flamingo, China, 2018 (MN917672.1)
Flamingo, China, 2018 (MN917673.1)
Freshwater shellfish, China, 2014 (NC_033050.1)
flactilig virus 13
Freshwater mussel, USA, 2018 (MT341478.1)
Freshwater shellfish, China, 2014 (NC_033087.1)
flactilig virus 16
Water sample, China, 2017 (MW348221.1)
Forsythia (plant), China, 2019 (MN823684.1)
Water sample, USA, 2010 (NC_043518.1)
Water sample, China, 2017 (MW347824.1)
Water sample, China, 2017 (MW347830.1)
Bee, Australia, 2013 (MG995716.1)
Water sample, China, 2017 (MW348238.1)
Freshwater mussel, USA, 2018 (MT341476.1)
flactilig virus 11
Water sample, China, 2017 (MW346750.1)
Bee, Australia, 2014 (MG995720.1)
Water sample, China, 2017 (MW346744.1)
Mantis shrimp, China, 2014 (NC_032553.1)
flactilig virus 15
Bee, Australia, 2014 (MG995708.1)
Water sample, USA, 2010 (NC_043519.1)
Water sample, USA, 2010 (NC_043515.1)
Freshwater snail, China, 2013 (NC_032963.1)
Ginko tree, China, 2019 (MN832466.1)
flactilig virus 12
flactilig virus 14
Water sample, China, 2017 (MW347539.1)
Freshwater snail, China, 2013 (NC_032917.1)
Lettuce, China, 2019 (MN832472.1)
Freshwater arthropod, China, 2013 (KX883647.1)
Red-crowned crane, China, 2018 (MT138371.1)
flactilig virus 4
Echinoderm, China, 2014 (NC_032529.1)
Millipedes, China, 2013 (NC_033733.1)
Giant river prawn, Malaysia, 2013 (KY607723.1)
Water sample, China, 2017 (MW347392.1)
Bivalve mollusc, China, 2017 (MG210792.1)
Asian hornet, France, 2014 (MN565031.1)
Drill, Nigeria, 2017 (MN296283.1)
Western honey bee, China, 2008 (HQ897161.1)
Midge, USA, 2016 (KX657788.1)
Fire ant, Argentina, 2014 (MH727530.1)
Abalone, Korea, 2017 (MG210797.1)
Abalone, Korea, 42736 (NC_040628.1)
Bird, China, 2018 (MN905999.1)
Bat, Republic of the Congo, 2018 (MH310078.1)
Horshoe crab, China, 2014 (KX883903.1)
Cricket, Thailand, 2013 (LC645209.1)
Insect, China, 2014 (KM270560.1)
Mosquito, France, 2014 (NC_030115.1)
Fly, Morocco, 2019 (MK645245.1)
Bee, Netherlands, 2014 (NC_035115.1)
flactilig virus 8
flactilig virus 7
flactilig virus 6
flactilig virus 5
Human, Australia, 2010 (KJ420970.1)
Human, Australia, 2010 (KJ420969.1)
Yellow-browed bunting, China, 2018 (MT138375.1)
Common blackbird, Belgium, 2017 (NC_040820.1)
clictilig virus 2
Penaeid shrimp, China, 2014 (NC_032589.1)
Freshwater arthropod, China, 2013 (NC_032809.1)
Freshwater snail, China, 2013 (NC_032921.1)
Razor shell, China, 2014 (NC_032576.1)
flactilig virus 9
Oyster, Italy, 2018 (MK561969.1)
Ant, United Kingdom, 2017 (NC_035457.1)
Ant, United Kingdom, 2017 (NC_035450.1)
Ant, Argentina, 2014 (MH727525.1)
Ant, Spain, 2014 (MH213247.1)
Bird, China, 2018 (MT137937.1)
Mosquito, Sweden, 2017 (MN513381.1)
Mosquito, South Korea, 2016 (NC_040584.1)
Spider, China, 2013 (NC_032222.1)
flactilig virus 10
0.3

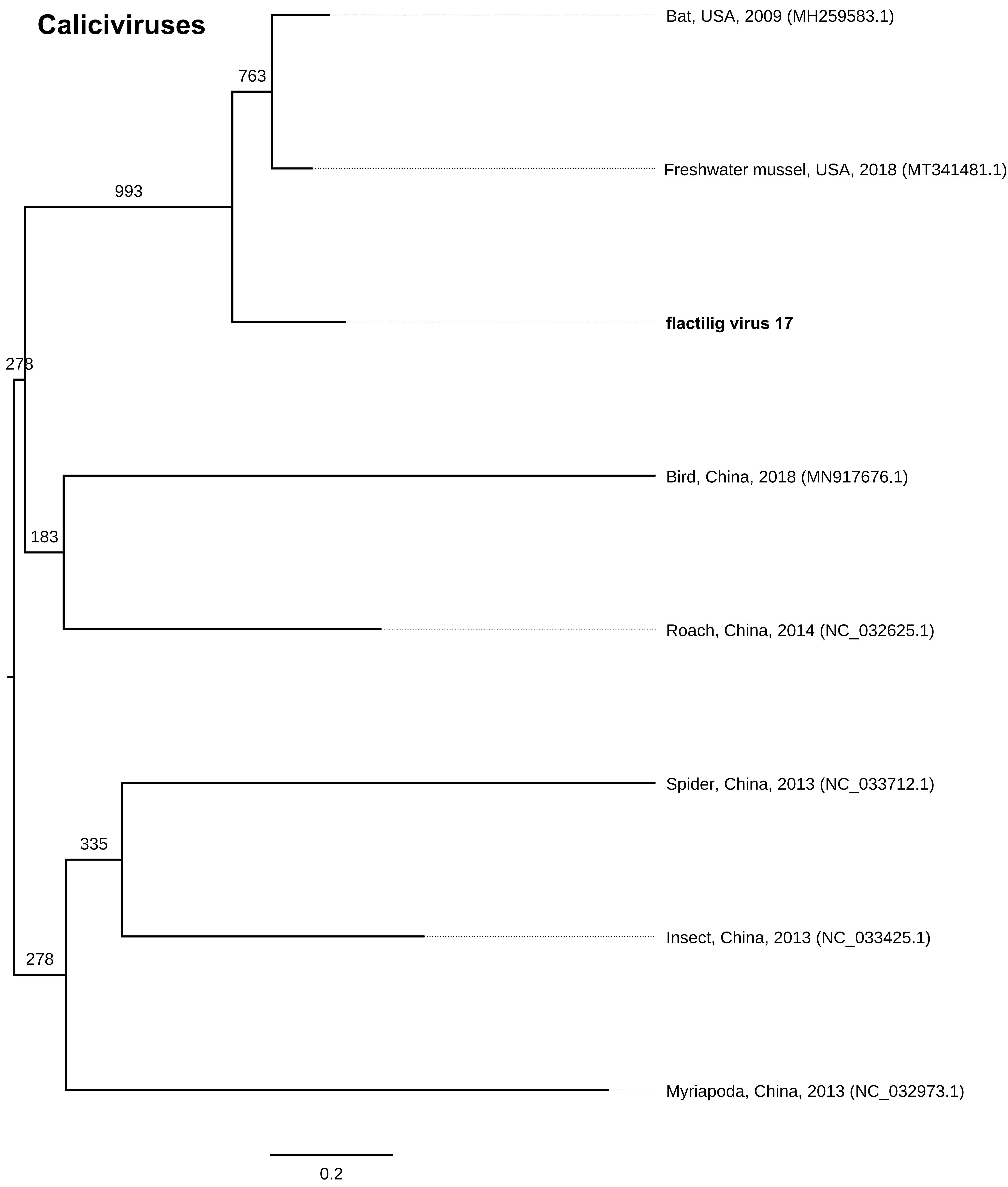
Caliciviruses
Bat, USA, 2009 (MH259583.1)
763
Freshwater mussel, USA, 2018 (MT341481.1)
993
flactilig virus 17
278
Bird, China, 2018 (MN917676.1)
183
Roach, China, 2014 (NC_032625.1)
Spider, China, 2013 (NC_033712.1)
335
Insect, China, 2013 (NC_033425.1)
278
Myriapoda, China, 2013 (NC_032973.1)
0.2

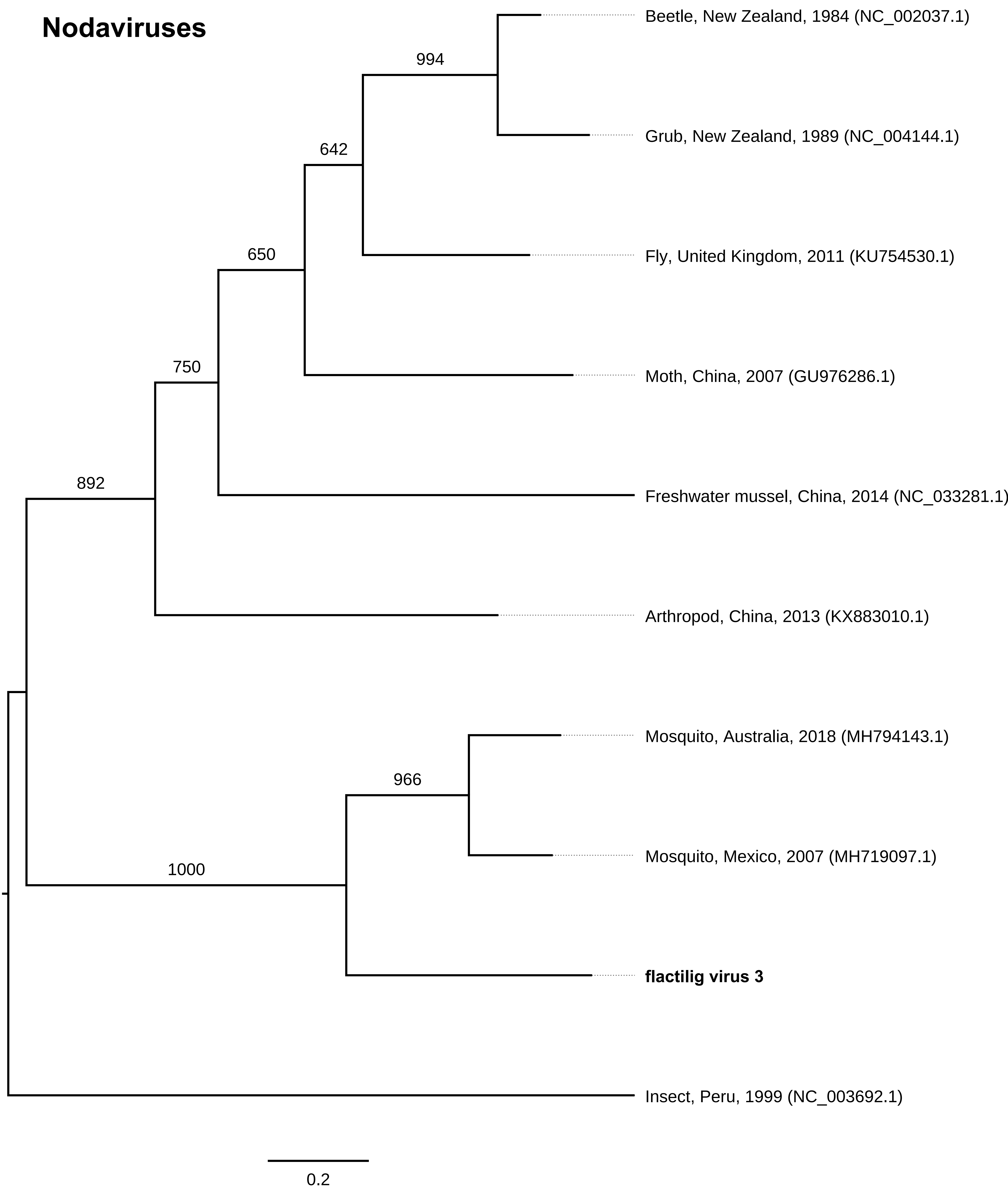
Nodaviruses
Beetle, New Zealand, 1984 (NC_002037.1)
Grub, New Zealand, 1989 (NC_004144.1)
Fly, United Kingdom, 2011 (KU754530.1)
Moth, China, 2007 (GU976286.1)
Freshwater mussel, China, 2014 (NC_033281.1)
Arthropod, China, 2013 (KX883010.1)
Mosquito, Australia, 2018 (MH794143.1)
Mosquito, Mexico, 2007 (MH719097.1)
flactilig virus 3
Insect, Peru, 1999 (NC_003692.1)
994
642
650
750
892
966
1000
0.2

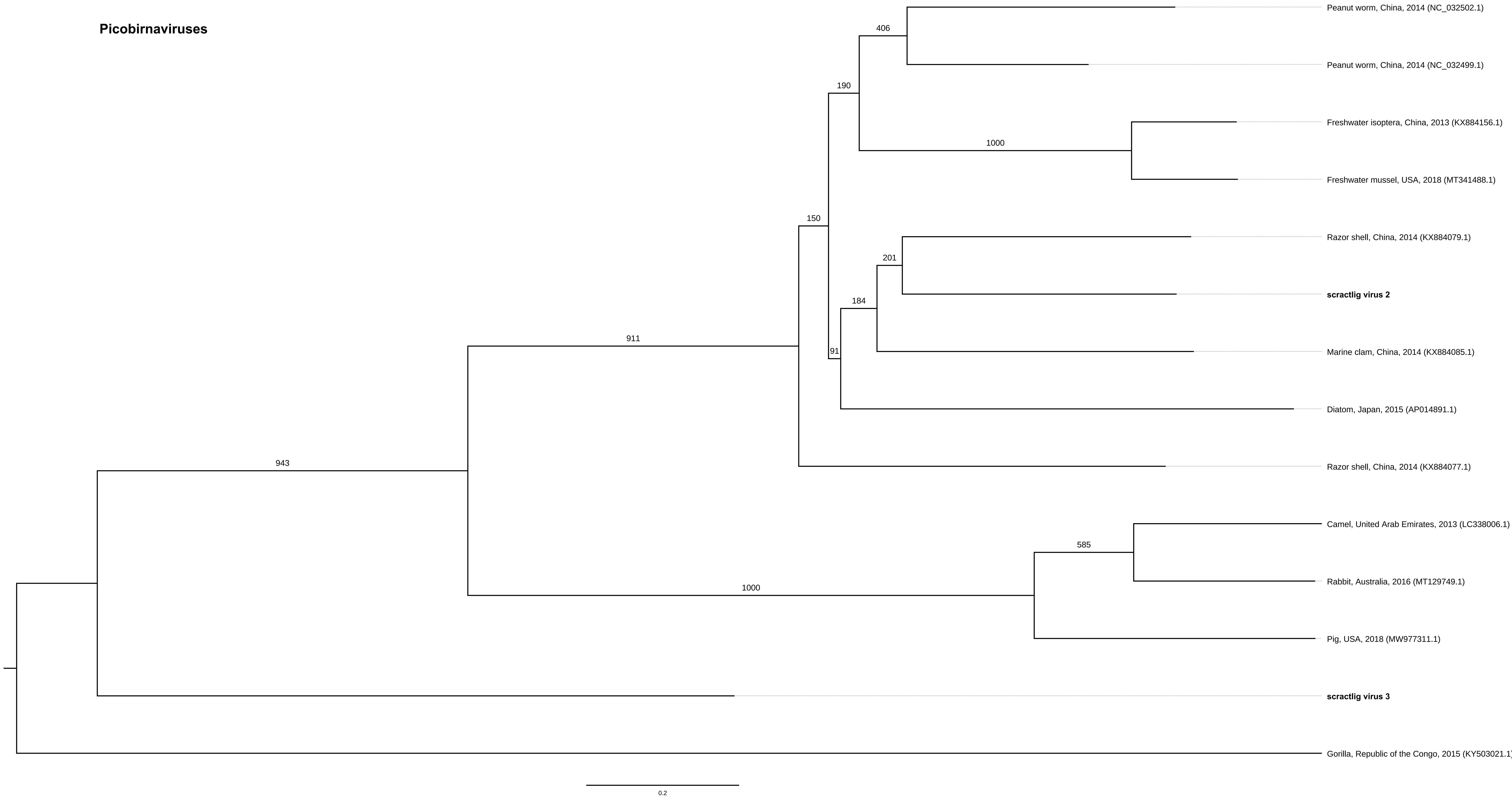

Picobirnaviruses
406
190
1000
150
201
184
911
91
943
585
1000
Peanut worm, China, 2014 (NC_032502.1)
Peanut worm, China, 2014 (NC_032499.1)
Freshwater isoptera, China, 2013 (KX884156.1)
Freshwater mussel, USA, 2018 (MT341488.1)
Razor shell, China, 2014 (KX884079.1)
scractlig virus 2
Marine clam, China, 2014 (KX884085.1)
Diatom, Japan, 2015 (AP014891.1)
Razor shell, China, 2014 (KX884077.1)
Camel, United Arab Emirates, 2013 (LC338006.1)
Rabbit, Australia, 2016 (MT129749.1)
Pig, USA, 2018 (MW977311.1)
scractlig virus 3
Gorilla, Republic of the Congo, 2015 (KY503021.1)
0.2

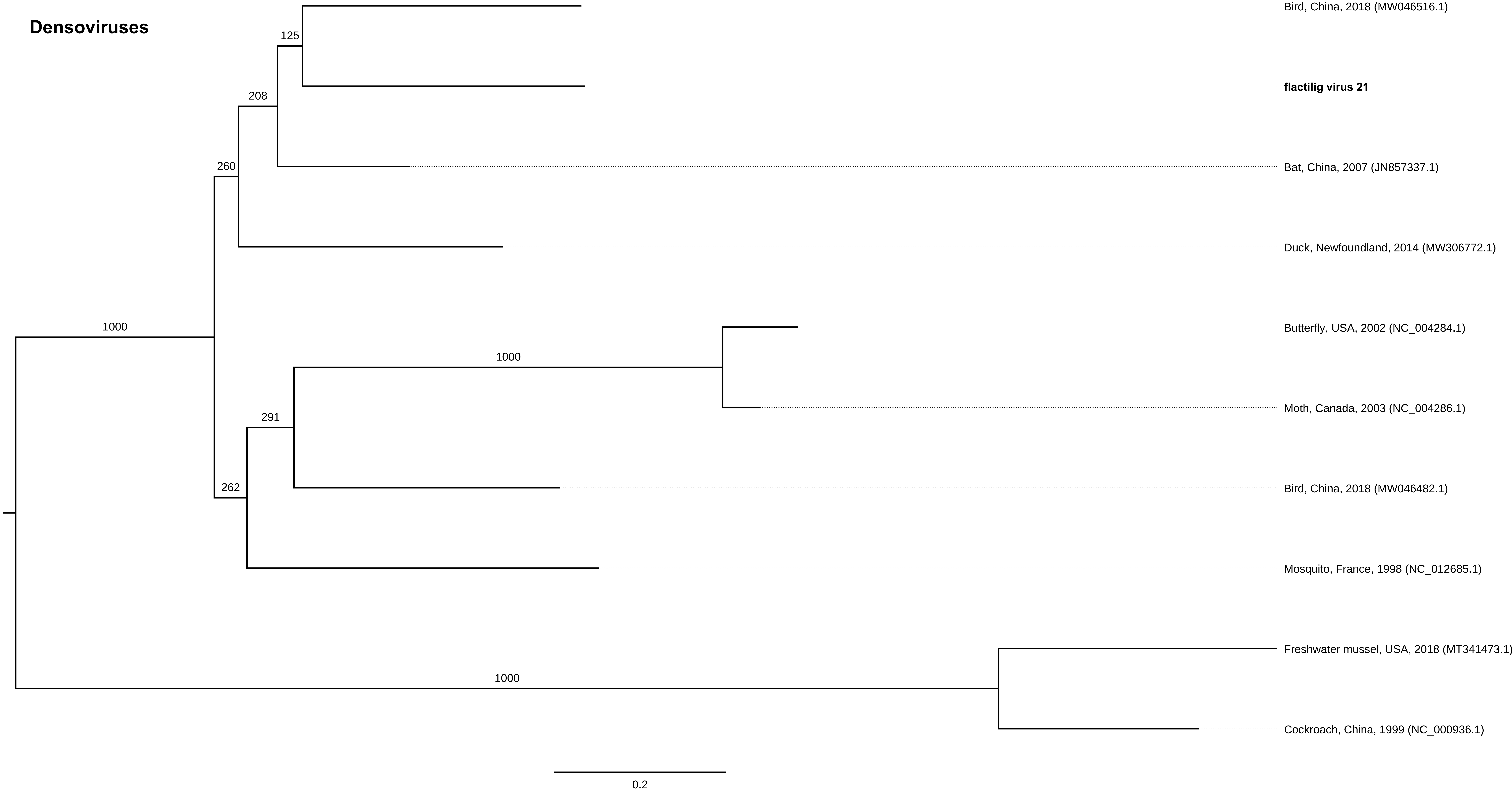
Densoviruses
Bird, China, 2018 (MW046516.1)
flactilig virus 21
Bat, China, 2007 (JN857337.1)
Duck, Newfoundland, 2014 (MW306772.1)
Butterfly, USA, 2002 (NC_004284.1)
Moth, Canada, 2003 (NC_004286.1)
Bird, China, 2018 (MW046482.1)
Mosquito, France, 1998 (NC_012685.1)
Freshwater mussel, USA, 2018 (MT341473.1)
Cockroach, China, 1999 (NC_000936.1)
125
208
260
1000
1000
291
262
1000
0.2

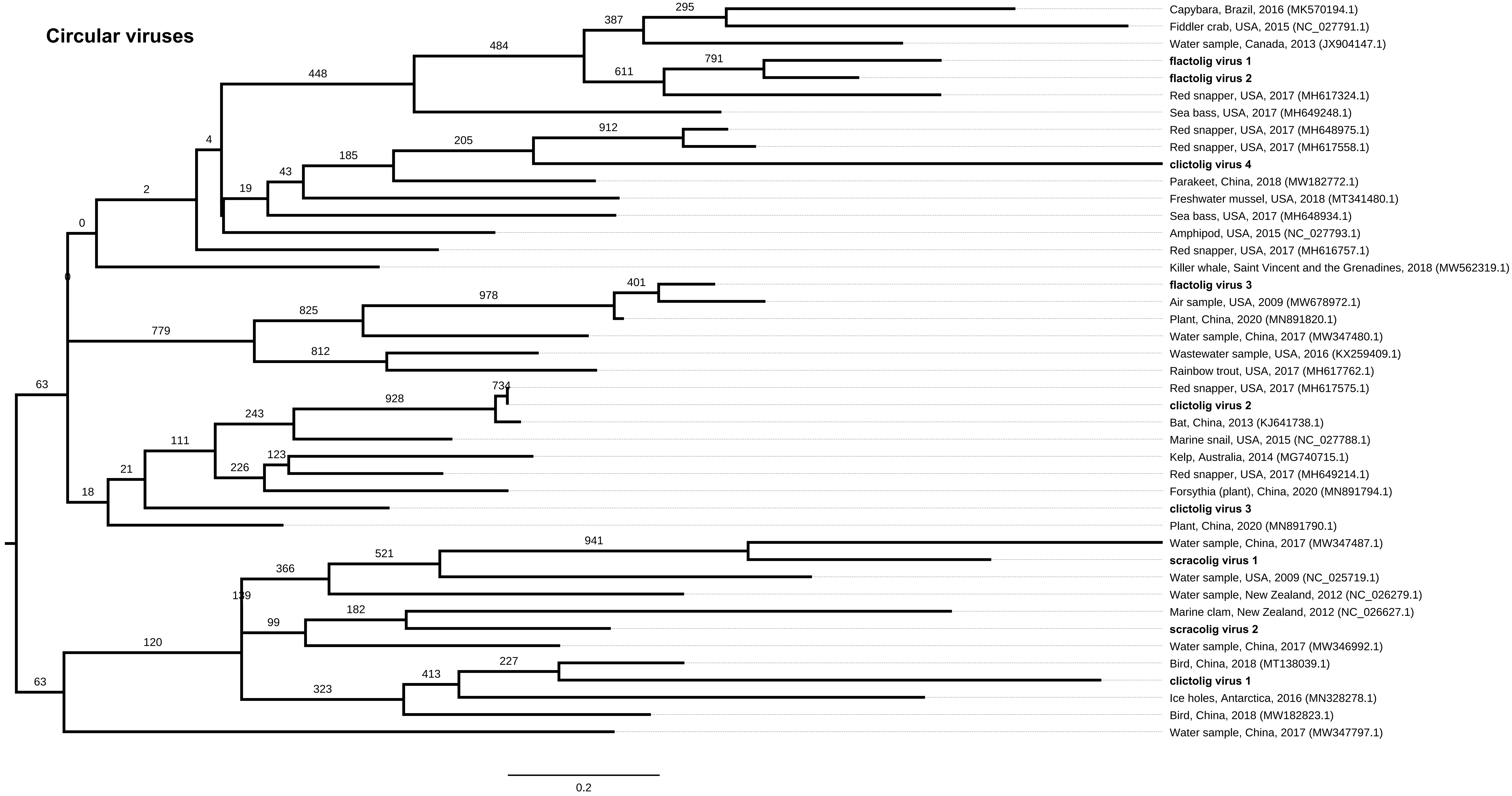
Circular viruses
Capybara, Brazil, 2016 (MK570194.1)
Fiddler crab, USA, 2015 (NC_027791.1)
Water sample, Canada, 2013 (JX904147.1)
flactolig virus 1
flactolig virus 2
Red snapper, USA, 2017 (MH617324.1)
Sea bass, USA, 2017 (MH649248.1)
Red snapper, USA, 2017 (MH648975.1)
Red snapper, USA, 2017 (MH617558.1)
clictolig virus 4
Parakeet, China, 2018 (MW182772.1)
Freshwater mussel, USA, 2018 (MT341480.1)
Sea bass, USA, 2017 (MH648934.1)
Amphipod, USA, 2015 (NC_027793.1)
Red snapper, USA, 2017 (MH616757.1)
Killer whale, Saint Vincent and the Grenadines, 2018 (MW562319.1)
flactolig virus 3
Air sample, USA, 2009 (MW678972.1)
Plant, China, 2020 (MN891820.1)
Water sample, China, 2017 (MW347480.1)
Wastewater sample, USA, 2016 (KX259409.1)
Rainbow trout, USA, 2017 (MH617762.1)
Red snapper, USA, 2017 (MH617575.1)
clictolig virus 2
Bat, China, 2013 (KJ641738.1)
Marine snail, USA, 2015 (NC_027788.1)
Kelp, Australia, 2014 (MG740715.1)
Red snapper, USA, 2017 (MH649214.1)
Forsythia (plant), China, 2020 (MN891794.1)
clictolig virus 3
Plant, China, 2020 (MN891790.1)
Water sample, China, 2017 (MW347487.1)
scracolig virus 1
Water sample, USA, 2009 (NC_025719.1)
Water sample, New Zealand, 2012 (NC_026279.1)
Marine clam, New Zealand, 2012 (NC_026627.1)
scracolig virus 2
Water sample, China, 2017 (MW346992.1)
Bird, China, 2018 (MT138039.1)
clictolig virus 1
Ice holes, Antarctica, 2016 (MN328278.1)
Bird, China, 2018 (MW182823.1)
Water sample, China, 2017 (MW347797.1)
0.2

Narnaviruses

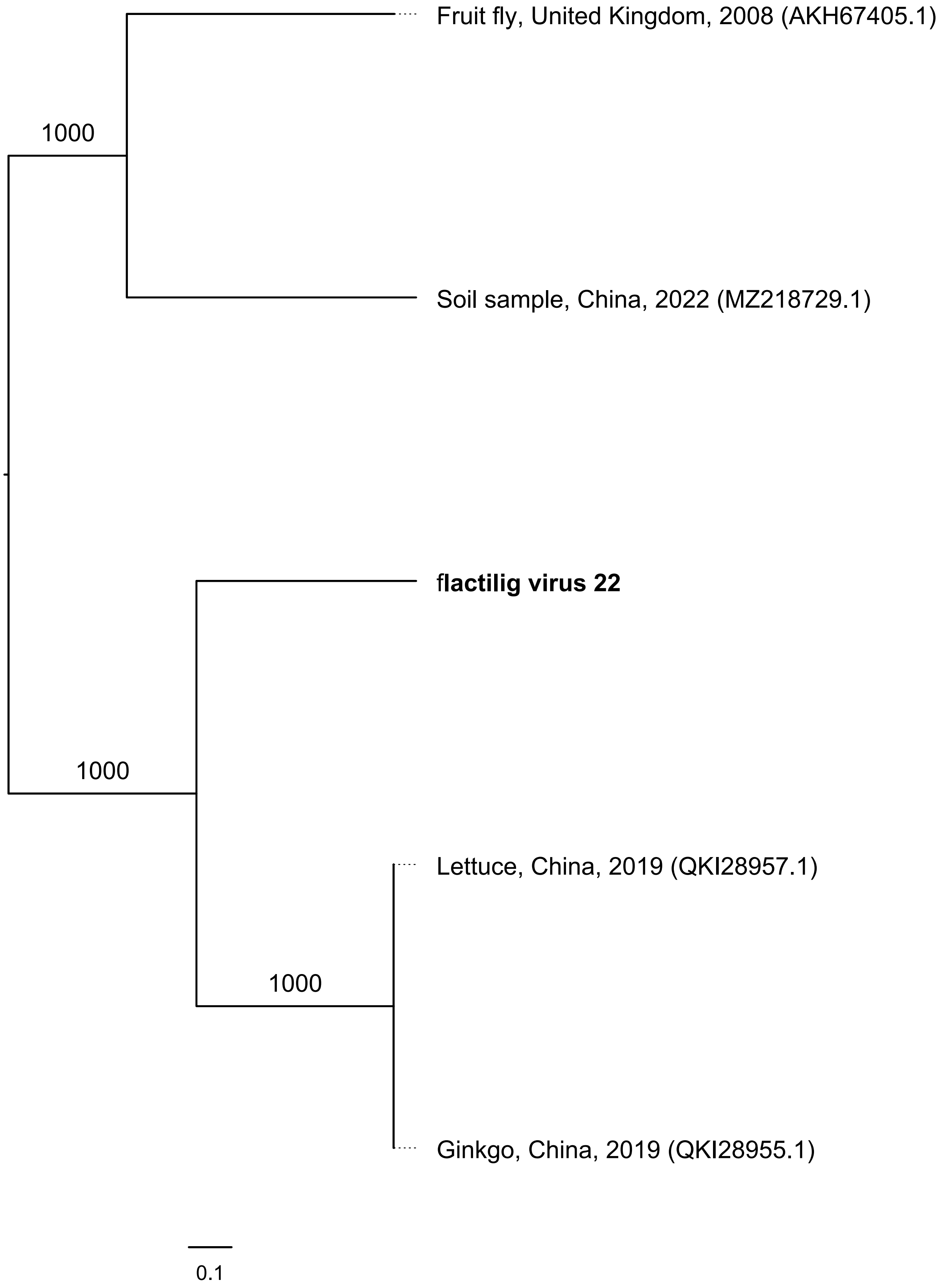

Reoviruses

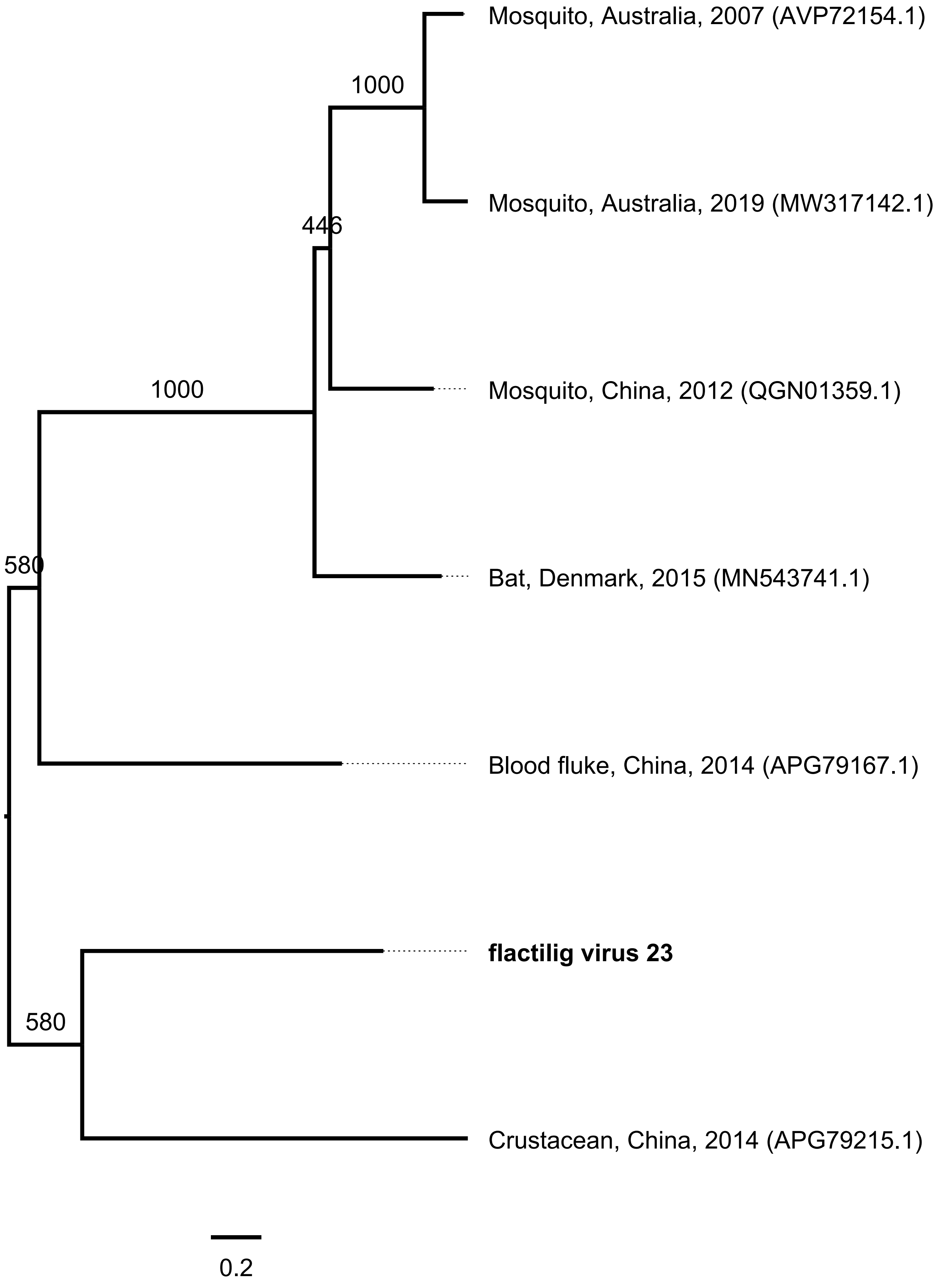

Mosquito, Australia, 2007 (AVP72154.1)
1000
Mosquito, Australia, 2019 (MW317142.1)
446
1000
Mosquito, China, 2012 (QGN01359.1)
580
Bat, Denmark, 2015 (MN543741.1)
Blood fluke, China, 2014 (APG79167.1)
flactilig virus 23
580
Crustacean, China, 2014 (APG79215.1)
0.2
