# Supplementary figures and images for: Freshwater Mussels Show Elevated Viral Richness and Intensity during a Mortality Event
Source: Viruses. 2022 Nov 23;14(12):2603. doi: 10.3390/v14122603 (PMC9785814; doi:10.3390/v14122603)

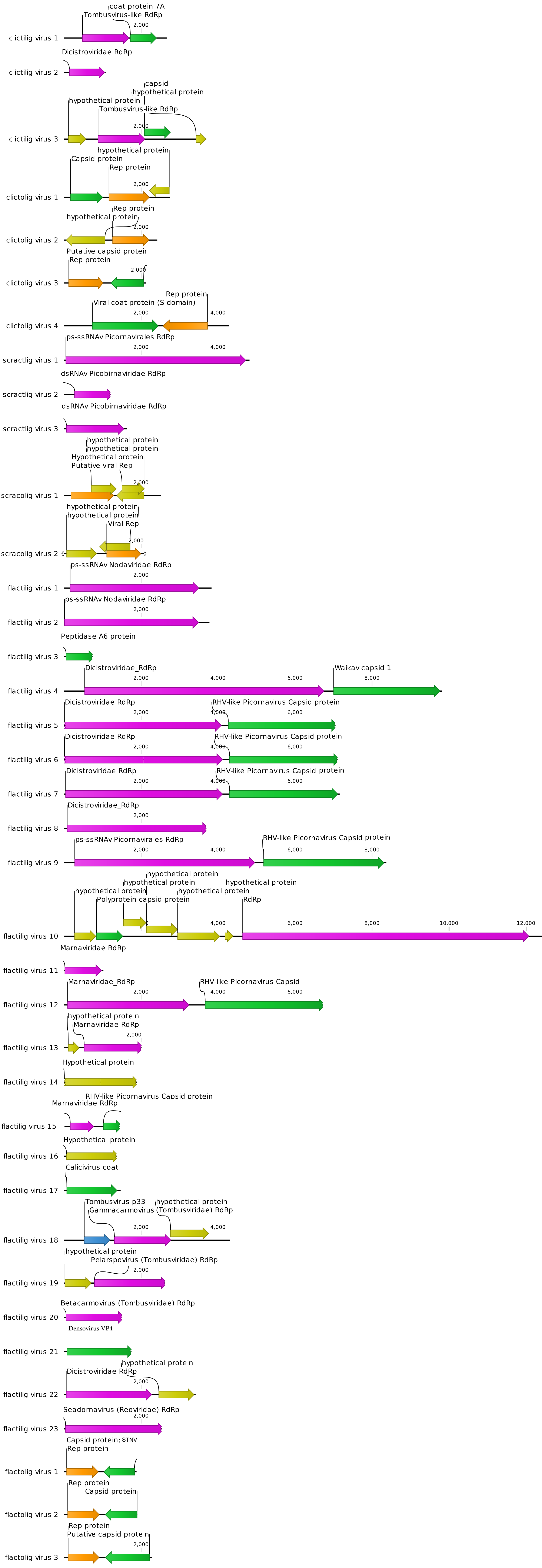

Supplement: Supplementary file 1 [file viruses-14-02603-s001.zip › Supplemental Figure S1 Mucket Virus Genome Maps.pdf]
